# Language and Sentiment Regarding Telemedicine and COVID-19 on Twitter: Longitudinal Infodemiology Study

**DOI:** 10.2196/28648

**Published:** 2021-06-21

**Authors:** Catherine C Pollack, Diane Gilbert-Diamond, Jennifer A Alford-Teaster, Tracy Onega

**Affiliations:** 1 Department of Biomedical Data Science Geisel School of Medicine at Dartmouth College Lebanon, NH United States; 2 Department of Epidemiology Geisel School of Medicine at Dartmouth College Lebanon, NH United States; 3 Department of Pediatrics Geisel School of Medicine at Dartmouth College Lebanon, NH United States; 4 Department of Medicine Geisel School of Medicine at Dartmouth College Lebanon, NH United States; 5 Department of Population Health Sciences Huntsman Cancer Institute University of Utah Salt Lake City, UT United States

**Keywords:** telemedicine, telehealth, COVID-19 pandemic, social media, sentiment analysis, Twitter, COVID-19, pandemic

## Abstract

**Background:**

The COVID-19 pandemic has necessitated a rapid shift in how individuals interact with and receive fundamental services, including health care. Although telemedicine is not a novel technology, previous studies have offered mixed opinions surrounding its utilization. However, there exists a dearth of research on how these opinions have evolved over the course of the current pandemic.

**Objective:**

This study aims to evaluate how the language and sentiment surrounding telemedicine has evolved throughout the COVID-19 pandemic.

**Methods:**

Tweets published between January 1, 2020, and April 24, 2021, containing at least one telemedicine-related and one COVID-19–related search term (“telemedicine-COVID”) were collected from the Twitter full archive search (N=351,718). A comparator sample containing only COVID-19 terms (“general-COVID”) was collected and sampled based on the daily distribution of telemedicine-COVID tweets. In addition to analyses of retweets and favorites, sentiment analysis was performed on both data sets in aggregate and within a subset of tweets receiving the top 100 most and least retweets.

**Results:**

Telemedicine gained prominence during the early stages of the pandemic (ie, March through May 2020) before leveling off and reaching a steady state from June 2020 onward. Telemedicine-COVID tweets had a 21% lower average number of retweets than general-COVID tweets (incidence rate ratio 0.79, 95% CI 0.63-0.99; *P*=.04), but there was no difference in favorites. A majority of telemedicine-COVID tweets (180,295/351,718, 51.3%) were characterized as “positive,” compared to only 38.5% (135,434/351,401) of general-COVID tweets (*P*<.001). This trend was also true on a monthly level from March 2020 through April 2021. The most retweeted posts in both telemedicine-COVID and general-COVID data sets were authored by journalists and politicians. Whereas the majority of the most retweeted posts within the telemedicine-COVID data set were positive (55/101, 54.5%), a plurality of the most retweeted posts within the general-COVID data set were negative (44/89, 49.4%; *P*=.01).

**Conclusions:**

During the COVID-19 pandemic, opinions surrounding telemedicine evolved to become more positive, especially when compared to the larger pool of COVID-19–related tweets. Decision makers should capitalize on these shifting public opinions to invest in telemedicine infrastructure and ensure its accessibility and success in a postpandemic world.

## Introduction

The COVID-19 public health crisis has transformed how individuals interact with critical services. This is particularly true of health care systems, which have been overwhelmed by patients with COVID-19 in both inpatient and intensive care units [1]. The sudden disruption in the ability to receive medical care has had widespread consequences for millions of Americans, and a survey by the US Centers for Disease Control and Prevention estimated that 40.9% of adults have delayed receiving medical care (including both emergency and routine care) due to concerns surrounding COVID-19 [2]. The new burdens placed on health care systems by the global pandemic have demonstrated the urgent need for the implementation of technologies to facilitate enhanced connectivity between patients and providers.

Telemedicine, defined as the delivery of health care services through electronic, audiovisual telecommunication systems, is not a novel concept—in fact, it has proven to be successful across a myriad of health domains [3]. The increased use of telemedicine during an emergency situation is also not new, and an increased uptake of telemedicine technologies has been observed during local, national, and international crises [4]. The implementation of telemedicine during a global pandemic is of particular value—not only can it be used to screen, diagnose, and triage patients from the comfort of their own homes, but it can also limit the need for physicians to use personal protective equipment that may be in low supply, facilitate rapid follow-up with diverse patient populations (particularly older patients or those living in a rural environment), reduce exposure to the infectious agent, and decrease the risk for intrahospital infection [5,6].

Despite its promise, prepandemic uptake of telemedicine in the United States was limited largely due to a lack of physician acceptance, stringent and heterogeneous licensing and reimbursement policies, and the upfront monetary costs of investing in the necessary infrastructure [4,5]. Nevertheless, shifts in policies in the peripandemic period have led to a surge in the uptake of telemedicine technologies. After the US Centers for Medicare & Medicaid Services revised their telemedicine reimbursement policies to include over 135 services (including emergency department visits, inpatient and nursing facility visits, and “discharge day management services”), over 36% of Medicare beneficiaries received at least one telemedicine service. Similarly, Medicaid and the Children’s Health Insurance Program reported a 2600% increase in the use of telemedicine services in March through June 2020 compared to the same period in 2019 [7]. Thus, there exists a clear demand for heterogeneous patient populations to receive care digitally. However, these utilization metrics may not directly correlate to patient satisfaction, willingness to engage in telemedicine, or their ability to do so. Thus, additional work is needed to quantify patients’ perceptions of the enhanced accessibility of telemedicine services.

Social media has increasingly been used as a surveillance tool by public health researchers to answer diverse health-related questions, including detecting disease outbreaks, situational awareness of humanitarian crises (such as natural disasters), and understanding a population’s reaction towards certain messaging or events [8]. This is particularly vital during an ongoing public health pandemic, wherein social media can provide insights more rapidly than traditional data collection methodologies such as surveys [9]. One of the more commonly used social media platforms for this purpose is Twitter, owing to the abundance of daily content and the widespread (although not necessarily representative) demographic reach of this platform [10]. A previous study evaluating telemedicine discourse on Twitter during the COVID-19 pandemic has identified distinct user networks that bridge content domains and user types (including educational, promotional, and political materials) [11], whereas another study found that the geographic distribution of telemedicine tweets in the United States was significantly correlated to the number of confirmed COVID-19 cases within a state [12]. However, both studies only evaluated a 1- to 2-week period, leaving a gap in understanding how conversations surrounding telemedicine may have changed over the course of the pandemic.

Furthermore, neither study considered the sentiment content of the tweets, which could provide more precise insights into how Twitter users perceive telemedicine. A separate evaluation of the sentiment of telemedicine-related tweets within a subpopulation of health care providers found overall positive opinions that focused on safety, accessibility, and implementation strategies [13]. However, because this study only focused on providers, there exists a dearth of research on the sentiment toward telemedicine expressed by the general Twitter population. Thus, the purpose of this study was to characterize how the content of telemedicine-related tweets has evolved during the COVID-19 pandemic, with a particular focus on changes in sentiment types. It was hypothesized that the frequency of telemedicine-related content on Twitter increased throughout the COVID-19 pandemic and that the sentiment of tweets became more positive across this period.

## Methods

### Data Collection

The characterization of telemedicine content in relation to COVID-19 was evaluated across 4 components of tweets: the number of favorites and retweets, language used within the tweet, sentiment of the tweet, and authorship. Tweets in English language posted between January 1, 2020, and April 24, 2021, containing at least one of a series of telemedicine-related terms and at least one of a series of COVID-19–related terms were curated from the Twitter full-archive search available via the Academic Research product track [14]. Search terms were derived from the literature and the Medical Subject Headings thesaurus established by the National Library of Medicine (NLM) of the National Institutes of Health (NIH) (Multimedia Appendix 1) [11,15]. A random sample of tweets containing only COVID-19–related terms was extracted for the same period for comparison with the distribution of sampled tweets matched with the daily distribution of telemedicine-related COVID-19 tweets. No geographic restrictions regarding the location of the tweet were implemented, and all tweets beginning with “RT @” (indicating retweet status) were removed prior to analysis.

### Text Processing

Standard natural language processing preprocessing procedures were performed prior to textual analysis of the tweets. First, links, mentions, hashtags, and HTML escape characters were removed from all tweets. Next, tweet-level sentiment was calculated using the Valence Aware Dictionary and sEntiment Reasoner (VADER), which was designed specifically for use on a social media corpus and has been validated in other Twitter-based studies, including one on telemedicine [16,17]. In addition to the compound sentiment score, a categorical sentiment was assigned to each tweet based on prespecified cutoffs within the literature (ie, *positive* if the compound sentiment was greater than 0.05, *negative* if the compound sentiment was lesser than –0.05, and *neutral* if the compound sentiment was between these values) [16]. After determining the sentiment, other preprocessing included tokenizing and lemmatizing the text and removing traditional English and Spanish stop words. In addition, words with an inverse document frequency in the 0.05th percentile were removed given their high frequency across all tweets (eg, “covid19”). Other words removed included non-English words (as determined by the GradyAugmented dataset [18]), as well as words with alternative connotations that may have skewed any analysis (eg, “trump” could refer to the verb or the 45th President of the United States; “patient” could refer to the adjective or a person receiving medical treatment). This processing was performed for both single words as well as bigrams (ie, two-word phrases) and trigrams (ie, three-word phrases).

### Author Analysis

In addition to evaluating all telemedicine-related COVID-19 tweets (“telemedicine-COVID”) relative to a general COVID-19 sample (“general-COVID”), the authors of tweets with the 100 most and the 100 fewest retweets were extracted and manually labeled with a domain (eg, “news,” “political,” “health and medicine”) and account type (ie, “organization” or “individual”). Domains were created by reviewing the author description information and, for verified users, confirming their identity through an independent Google search. Individual authors who represented a nonverified person were automatically labeled as “private citizen,” whereas all organizations were labeled with a domain regardless of the verification status. This process was completed by one member of the research team for both the telemedicine-COVID and general-COVID data sets to compare outcomes within these tweet subpopulations. A second member of the research team independently labeled 10% of the authors as a “validity check” [19]. This subsequentially resulted in Cohen κ=0.66 (percent agreement: 77.3%) for domain type and κ= 0.75 (percent agreement: 86.4%) for the account type.

### Statistical Analysis

A comparative analysis of retweets and favorites by month and by data set (ie, telemedicine-COVID vs general-COVID) was conducted using zero-inflated Poisson regression to accommodate for the preponderance of tweets receiving no favorites or retweets and the count nature of the outcome variable. Sentiment analysis was also performed to assess the distribution of positive, negative, and neutral tweets both overall and by month and compared using chi-square tests. This analysis was repeated to compare sentiment in the telemedicine-COVID data set with that in the general-COVID data set. Chi-square tests were also used to compare the distribution of sentiment between the top 100 most and least retweeted posts both within each data set and between them. All analyses were conducted in Python (version 3.7.4) within the Jupyter Notebook graphical user interface (GUI) (version 7.19.0) and R (version 3.6.3) within the RStudio GUI (version 1.3.959). Code is available on GitHub [20]; tweet IDs are available upon request. Given the public nature of social media data, institutional review board approval was not required as specified in Regulation 45 CFR 46 as authored by the US Department of Health and Human Services Office for Human Research Protections [21].

## Results

After removing duplicate tweets and retweets from analysis, 351,718 tweets related to telemedicine and COVID-19 were used in the analytic data set (telemedicine-COVID). A sample comparator data set (general-COVID) of 351,401 tweets was collected with a similar daily distribution for comparison.

**Figure 1:**
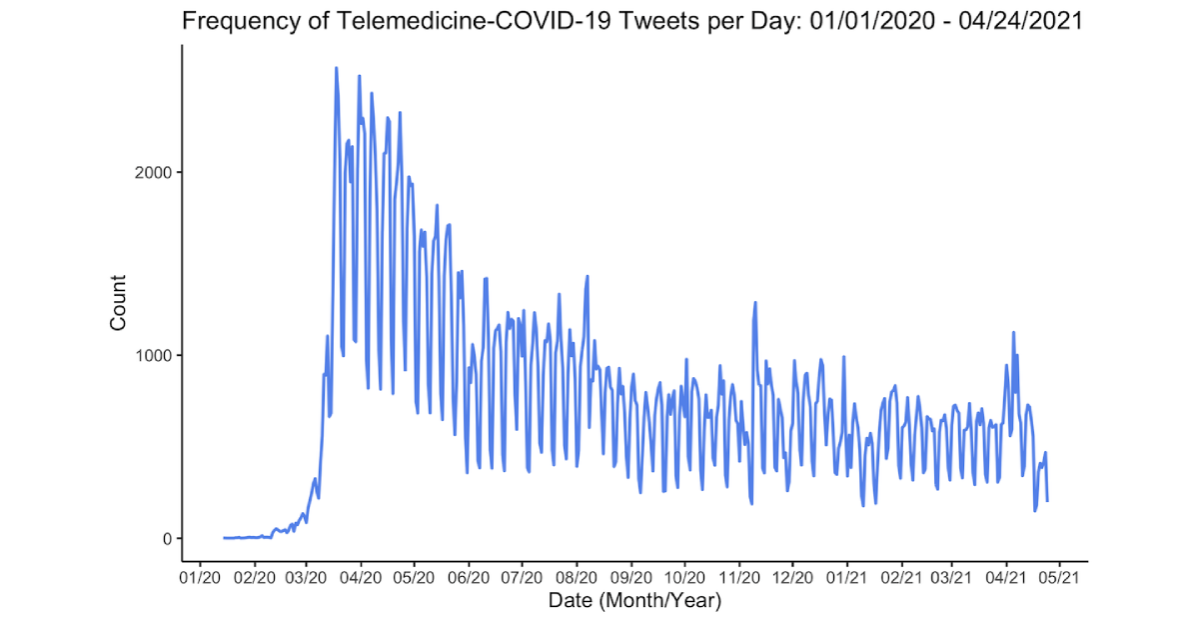
Frequency of tweets mentioning telemedicine and COVID-19 between January 1, 2020, and April 24, 2021 (N=351,718).

**Figure 2:**
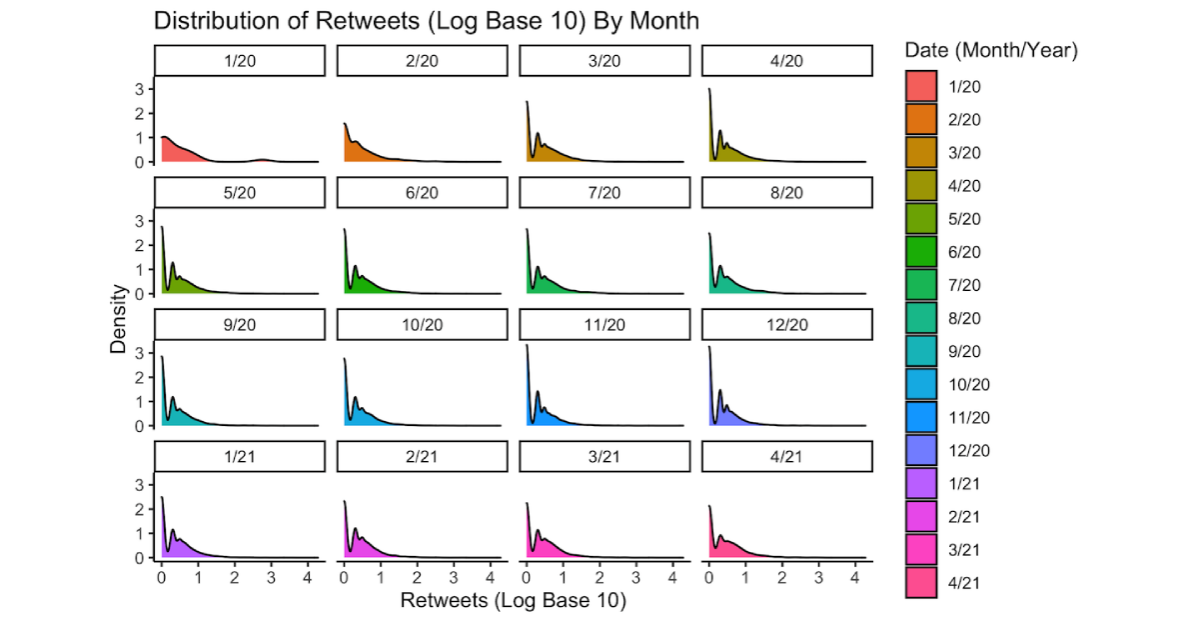
Monthly variations in the base 10 log number of retweets for tweets mentioning COVID-19 and telemedicine. Note that 219,212 tweets (62.2%) had no retweets and are not included in this visualization.

Telemedicine-COVID tweets spiked in the early stages of the pandemic (eg, March 2020 to May 2020) before leveling off in the following months (Figure 1). There were significant monthly variations in the number of retweets of telemedicine-COVID tweets (Figure 2). Retweets peaked in March 2020, with April, May, July, September, October, and December 2020 and January through April 2021 having significantly fewer retweets by comparison (Table S1 in Multimedia Appendix 2). Tweets from the telemedicine-COVID data set had a 21% lower average number of retweets than tweets from the general-COVID data set (incidence rate ratio [IRR] 0.79, 95% CI 0.63-0.99; *P*=.04; Table S2 in Multimedia Appendix 2). In terms of favorites, telemedicine-COVID tweets in April, May, July, August, September, October, and December 2020, and tweets from January through April 2021 had a significantly lower average number of favorites than those in March 2020 (Table S3 in Multimedia Appendix 2). Telemedicine-COVID tweets had a 14% lower average number of favorites than did general-COVID tweets, but this value was not statistically significant (IRR 0.86, 95% CI 0.58-1.28; *P*=.45) (Table S4 in Multimedia Appendix 2).

**Figure 3:**
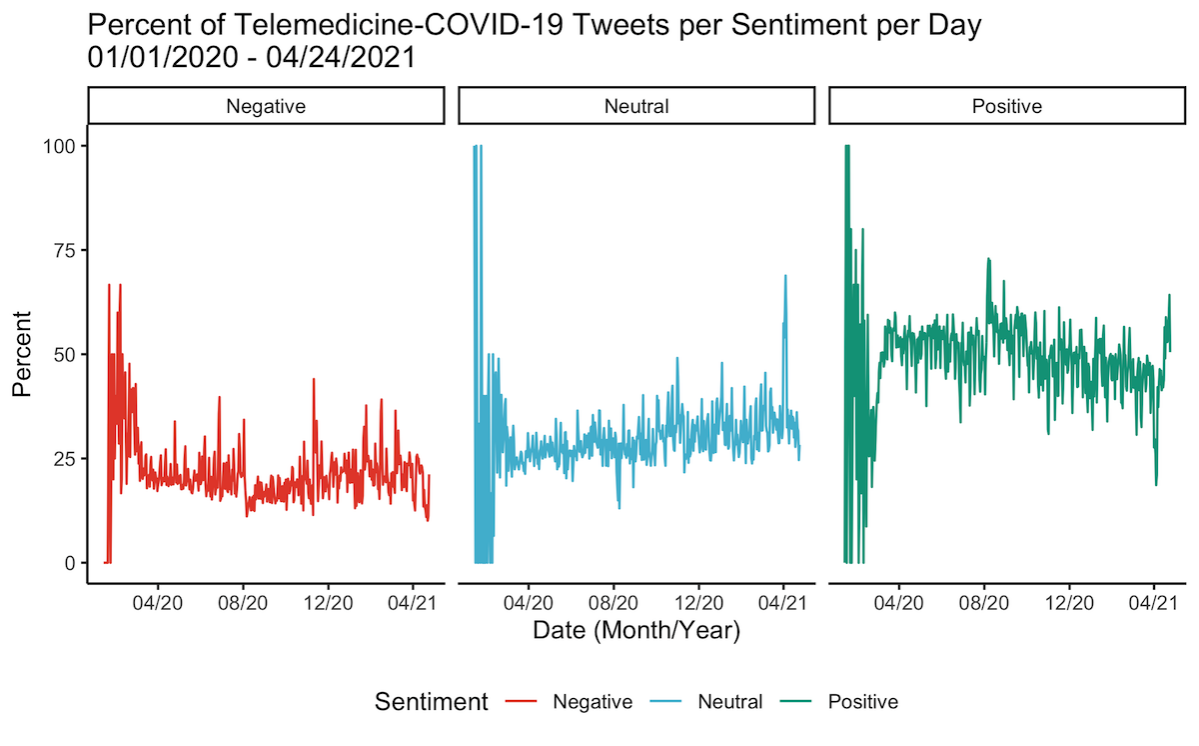
Changes in the frequency of positive, negative, and neutral tweets mentioning telemedicine and COVID-19 posted between January 1, 2020, and April 24, 2021 (N=351,718).

**Figure 4:**
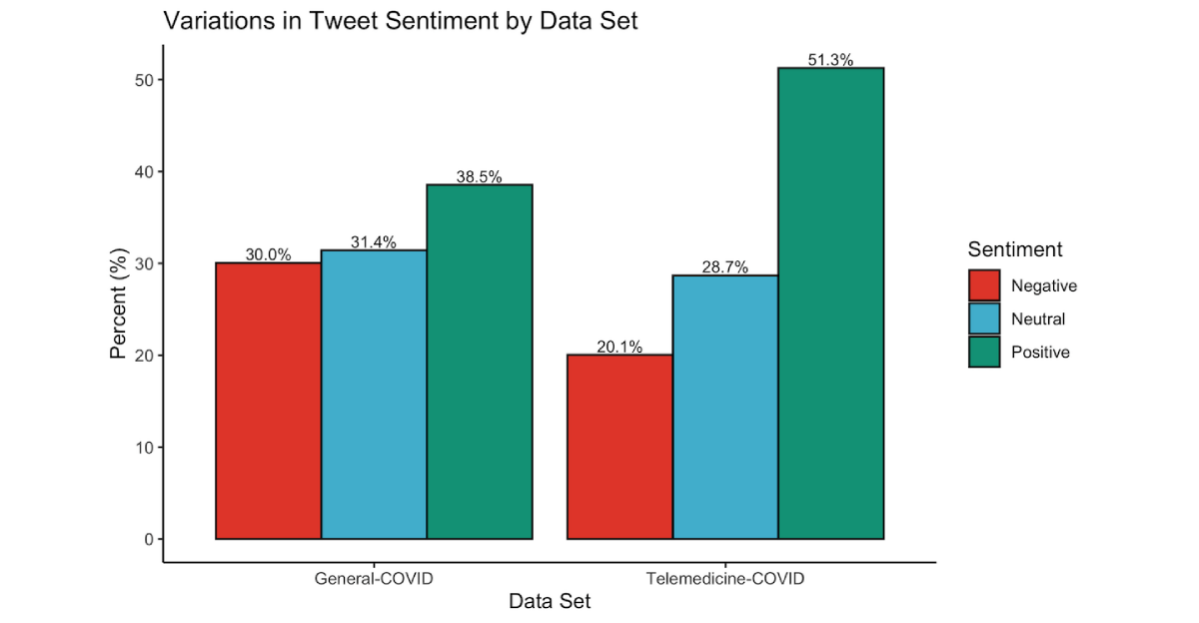
Variations in sentiment by tweet type (ie, telemedicine and COVID-19 vs COVID-19 only). A significantly higher proportion of tweets with a positive sentiment was found in the telemedicine-COVID data set than in the general-COVID data set (*P*<.001).

When broken down by sentiment, the percentage of telemedicine-COVID tweets with an overall sentiment of positive or neutral generally increased over the period of interest, whereas the percentage of negative tweets decreased (Figure 3). There were significant monthly variations in the percentage of tweets with each sentiment (*P*<.001). The months with the highest percentage of positive telemedicine-COVID tweets were August 2020 (14,371/24,543, 58.6%), September 2020 (10,475/18,758, 55.8%), and March 2020 (19,851/36,478, 54.4%), whereas the months with the highest percentage of negative tweets were February 2020 (453/1274, 35.6%), January 2020 (13/43, 30.2%), and January 2021 (3695/16,613, 22.2%). There were also significant differences in the distribution of positive, negative, and neutral tweets between the telemedicine-COVID and general-COVID data sets (*P*<.001; Figure 4). Although sentiments were evenly distributed among general-COVID tweets (38.5% positive, 31.4% neutral, and 30.0% negative), a majority of telemedicine-COVID tweets (N=351,718) were positive (n=180,295, 51.3%), followed by neutral (n=100,870, 28.7%). There were also significant variations between data sets on a month-to-month basis for March 2020 through April 2021, with a significantly higher proportion of positive tweets in the telemedicine-COVID data set relative to the general-COVID data set (Multimedia Appendix 3).

Within the telemedicine-COVID tweet data set (N=351,718), the most frequently used bigrams were “town hall” (n=1411, 0.40%); “white house” (n=848, 0.24%), “san diego” (n=757, 0.22%), “mask wearing” (n=670, 0.19%), “lessons learned” (n=610, 0.17%), and “artificial intelligence” (n=562, 0.16%). The most frequently used trigrams were “widespread mask wearing” (n=385, 0.11%), “feeling helpless hopeless” (n=301, 0.09%), “fast track vital” (n=199, 0.06%), “cancer sooner simply” (n=191, 0.05%), “thermal imaging cameras” (n=188, 0.05%), and “list refresh page” (n=176, 0.05%).

In contrast, within the general-COVID sample (N=351,401), the most frequently used bigrams were “ill [*sic*] deliver” (n=1195, 0.34%), “nursing homes” (n=693, 0.20%), “task force” (n=597, 0.17%), “herd immunity” (n=563, 0.16%), “prime minister” (n=508, 0.14%), and “town hall” (n=439, 0.12%). The most frequently used trigrams were “appointment detected provider” (n=214, 0.06%), “personal protective equipment” (n=205, 0.06%), “complete project chapter” (n=181, 0.05%), “wall street journal” (n=101, 0.03%), “operation warp speed” (n=68, 0.02%), and “midnight mm rain” (n=67, 0.02%). A word cloud of unigrams is presented in Multimedia Appendix 4.

When broken down by authorship, the top 100 most retweeted telemedicine-COVID tweets were predominantly authored by politicians (20/93, 21.5%) and private citizens (20/93, 21.5%), followed by journalists (14/93, 15.1%). Similarly, the top 100 most retweeted general-COVID sample tweets were predominantly authored by private citizens (23/89, 25.8%), journalists (16/89, 18.0%), and politicians (13/89, 14.6%). Of the sample of tweets without any retweets, 82.8% (72/87) of those from the general-COVID sample and 61.1% (55/90) from the telemedicine-COVID sample were authored by private citizens. There was a small subset of authors within each data set that did not have available account information (7 authors of the top 100 telemedicine-COVID retweets, 10 authors of the bottom 100 telemedicine-COVID retweets, 11 authors of the top 100 general-COVID retweets, and 13 authors of the bottom 100 general-COVID retweets). In terms of sentiment, of the most retweeted tweets within the telemedicine-COVID data set, 54.5% (55/101) were positive and 30.7% (31/101) were negative. In contrast, of the fewest retweeted tweets, 51.3% (40/78) were positive and 19.2% (15/78) were negative (*P*=.03). Within the general-COVID data set, 49.4% (44/89) of the top retweeted tweets were negative and 33.7% (30/89) were positive. This was not significantly different from the fewest retweeted general-COVID posts (*P*=.16), but it was significantly different from the most retweeted telemedicine-COVID tweets (*P*=.01). The sentiment of the fewest retweeted general-COVID did not significantly differ from the sentiment of the fewest retweeted telemedicine-COVID tweets (*P*=.07).

## Discussion

### Principal Findings

To the best of our knowledge, this study is the first to comprehensively evaluate tweets pertaining to telemedicine and COVID-19 posted between January 2020 and April 2021 compared to a general COVID-19 data set. Analysis of retweets and favorites suggested monthly variations in the “attention” received by telemedicine-COVID tweets, and these tweets had a significantly lower average number of retweets than the general-COVID data set. Telemedicine-COVID tweets were predominantly positive both overall and by month, especially compared to the general-COVID data set. There were also variations in the frequency and sentiment of tweets made by entities in various domains, including private citizens, politicians, and journalists, and a higher proportion of the most retweeted tweets in the telemedicine-COVID data set were positive than those in the general-COVID data set. The findings presented here demonstrate how social media can be leveraged to perform surveillance of shifting opinions surrounding critical health technologies, including telemedicine.

The number of tweets that mentioned telemedicine and COVID-19 drastically increased between February and March 2020, coinciding with the declaration of the COVID-19 pandemic by the World Health Organization on March 11, 2020 [22]. As stay-at-home orders continued throughout April and May 2020, telemedicine became more prominent within the COVID-19 dialogue on Twitter, and health systems began to adapt to the use of this technology. Discussions surrounding telemedicine decreased through June 2020 and remained relatively constant thereafter, perhaps reflecting the achievement of “steady state.” This leveling off coincides with previous studies that have found that the weekly rate of telemedicine consults in a Medicare population peaked in April before declining in June 2020 [23].

As the volume of telemedicine-COVID tweets evolved, so did the sentiment of these tweets. Over half of all months between March 2020 and April 2021 had a majority of telemedicine-COVID tweets labeled as positive, and all months during this period had a significantly higher proportion of positive tweets compared to the general-COVID data set. Although this finding aligns with a study prior to the pandemic that found a higher number of positive telemedicine tweets than negative and neutral tweets, it contrasts with another study that found that 59% of individuals were either unsure of telemedicine or considered it subpar to traditional care delivery mechanisms [17,24]. The positive results found in this study may be reflective of increasing acceptance toward telemedicine—while patients may have viewed telemedicine as just an alternative to in-person care before the pandemic, telemedicine may now be viewed as an alternative to no care whatsoever. This finding aligns with recent patient surveys on their opinion of telemedicine, which found that 79% of patients were “satisfied” with their experience with telemedicine and 78% felt that they had a health problem that could be addressed virtually [25]. Furthermore, increased positivity in the middle stages of the pandemic (particularly in August and September 2020) reflects the evolving understanding of the Twitter population that reduced contact during a telemedicine visit can provide a safer experience during a highly contagious disease outbreak. The increase in positivity in this period in particular also corresponds to the signing of the Executive Order 13941 on August 3, 2020, which aimed to “improve rural health and telehealth access” to Medicare beneficiaries during the postpandemic period [26,27].

The most followed accounts within both the telemedicine-COVID and general-COVID data sets predominantly consisted of journalists and politicians. These groups present stark contrasts in content veracity—journalists likely provide neutral content with minimal bias (depending on the agency), whereas politicians likely share more polarized content that reflect their own views. Prior work evaluating tweets on telemedicine during the COVID-19 pandemic (but not necessarily related to it) found that private citizens frequently retweeted content from both sources, although politicians were retweeted more frequently [11]. Thus, there is a chance that a larger number of Twitter users’ opinions on telemedicine and COVID-19 may be limited to “echo chambers” that reinforce their own opinion and, in a worst-case scenario, spread misinformation with deleterious consequences. However, the fact that a majority of tweets within the telemedicine-COVID data set were positive suggests that this may not necessarily be the case for tweets pertaining to telemedicine or other novel technologies. Conjecturally, this may reflect that positive news and experiences on telemedicine were amplified during the COVID-19 pandemic, which could lead to more widespread adoption and uptake of this technology.

### Policy Implications

Telemedicine is the pinnacle “21st century approach” to deliver convenient and less expensive care, and over 50 large US health systems have integrated it into their standard operating procedures [5]. Although the surge in telemedicine visits during the early phases of the pandemic have waned to some extent, evidence shows a high (but geographically variable) degree of persistence of telemedicine, from 8% to almost 48% [23]. This can likely be attributed to relaxations in policies that presented major challenges to telemedicine uptake, including reimbursement parity, interstate licensing, prescribing practices, the use of Health Insurance Portability and Accountability Act (HIPAA)-compliant technologies, and the definition of an “originating site” (ie, where the patient is located), to name a few [28,29]. The overwhelming and consistent positive nature of conversations surrounding telemedicine on Twitter as presented here, coupled with a surge in uptake throughout the pandemic, demonstrate that diverse, heterogeneous populations view telemedicine favorably, including patients, providers, and politicians. These findings provide clear evidence for policymakers that replacing restrictive policies with long-term, systematic favorable toward telemedicine would be met with support from numerous, diverse communities.

### Limitations and Future Directions

Despite the promise of this study, it is not without its limitations. Although the list of search terms was comprehensive and based on prior literature, it may have missed tweets that used other terminology to describe telemedicine-related services. Similarly, it is possible that some of the included tweets may not have directly been related to telemedicine. The analysis also does not include telemedicine-related tweets that were made during the study period that did not contain a direct mention of COVID-19. Although this ensured that the data specifically focused on the impacts of telemedicine as they related to COVID-19, future work could analyze how telemedicine tweets that directly mentioned COVID-19 varied from those that did not during the same time. Furthermore, future work could evaluate whether the trends observed in the varied from tweets made during the same period in the prior year. In addition, the present work does not include geospatial data, which has previously been shown to be an influential component of tweet sentiment [30,31]. Thus, future work could evaluate geospatial variations in telemedicine sentiment, including whether it is associated with uptake of the technology in local medical facilities. In addition, only the top 100 most and least retweeted posts were included in the author-level analysis, and future work could expand on this to label more accounts, improve the classification of labels (eg, labeling fewer accounts as “private citizens”), or analyze only a subset of these accounts (eg, politicians or “influencers”). Lastly, the Twitter population of predominantly 18-24 years old, well-educated individuals is not representative of a US or broader population [32]. Thus, future work is needed to characterize the change in sentiment within other populations, especially those that may not be technologically literate and may therefore encounter barriers when attempting to utilize telemedicine.

### Conclusions

Opinions on telemedicine and COVID-19 on Twitter have increased in popularity and were largely positive throughout 2020 and the beginning of 2021. These telemedicine-COVID tweets were generally more positive than general-COVID tweets both overall and within the subset of the most followed authors, suggesting an amplification of discussion surrounding the benefits of telemedicine. Given the relative positivity with which individuals seemed to view telemedicine during the COVID-19 pandemic, shifts in policies stemming from the COVID-19 pandemic that support telemedicine are likely to be well received.

## Appendix

Multimedia Appendix 1 Telemedicine and COVID-19–related search terms used to select tweets.

Multimedia Appendix 2 Zero-inflated Poisson models.

Multimedia Appendix 3 Sentiment variations between the telemedicine-COVID data set and the general-COVID data set by month.

Multimedia Appendix 4 Word clouds for unigrams for telemedicine-specific COVID-19 tweets and general COVID-19 sample tweets.

## Abbreviations

HIPAA: Health Insurance Portability and Accountability Act
GUI: graphical user interface
IRR: incidence risk ratio
NCI: National Cancer Institute
NIH: National Institutes of Health
NLM: National Library of Medicine
VADER: Valence Aware Dictionary and sEntiment Reasoner

## References

[1] Shammas B, Cha A, Guarino B, Dupree J (2020). Record numbers of covid-19 patients push hospitals and staffs to the limit. Washington Post.

[2] Czeisler ME, Marynak K, Clarke KEN, Salah Z, Shakya I, Thierry JM, Ali N, McMillan H, Wiley JF, Weaver MD, Czeisler CA, Rajaratnam SMW, Howard ME (2020). Delay or avoidance of medical care because of COVID-19-related concerns - United States, June 2020. MMWR Morb Mortal Wkly Rep.

[3] Ekeland AG, Bowes A, Flottorp S (2010). Effectiveness of telemedicine: a systematic review of reviews. Int J Med Inform.

[4] Smith AC, Thomas E, Snoswell CL, Haydon H, Mehrotra A, Clemensen J, Caffery LJ (2020). Telehealth for global emergencies: implications for coronavirus disease 2019 (COVID-19). J Telemed Telecare.

[5] Hollander JE, Carr BG (2020). Virtually perfect? telemedicine for Covid-19. N Engl J Med.

[6] Bokolo AJ (2021). Exploring the adoption of telemedicine and virtual software for care of outpatients during and after COVID-19 pandemic. Ir J Med Sci.

[7] (2020). Trump administration drives telehealth services in Medicaid and Medicare. Centers for Medicare & Medicaid Services.

[8] Fung IC, Tse ZTH, Fu K (2015). The use of social media in public health surveillance. Western Pac Surveill Response J.

[9] Murray CJL, Alamro NMS, Hwang H, Lee U (2020). Digital public health and COVID-19. Lancet Public Health.

[10] Sinnenberg L, Buttenheim AM, Padrez K, Mancheno C, Ungar L, Merchant RM (2017). Twitter as a tool for health research: a systematic review. Am J Public Health.

[11] Lit CL, Khayal IS (2020). Understanding Twitter telehealth communication during the COVID-19 pandemic using hetero-functional graph theory.

[12] Massaad E, Cherfan P (2020). Social media data analytics on telehealth during the COVID-19 pandemic. Cureus.

[13] Larson S, Popov V, Ali A, Ramanathan P, Jung S, Ruis AR, Lee SB (2021). Healthcare professionals’ perceptions of telehealth: analysis of tweets from pre- and during the COVID-19 pandemic. Advances in Quantitative Ethnography. ICQE 2021. Communications in Computer and Information Science, vol 1312.

[14] Academic research - Preparing for the application. Twitter Developer Platform.

[15] MeSH (Medical Subject Headings). National Center for Biotechnology Information.

[16] Hutto CJ, Gilbert E (2014). VADER: a parsimonious rule-based model for sentiment analysis of social media text.

[17] Talpada H, Halgamuge MN, Tran Quoc Vinh N (2019). An analysis on use of deep learning and lexical-semantic based sentiment analysis method on Twitter data to understand the demographic trend of telemedicine.

[18] Rinker T GradyAugmented: augmented list of Grady Ward's English words and Mark Kantrowitz's names list. RDocumentation.

[19] Buchanan L, Yeatman H, Kelly B, Kariippanon K (2018). A thematic content analysis of how marketers promote energy drinks on digital platforms to young Australians. Aust N Z J Public Health.

[20] Telemedicine and COVID-19. GitHub.

[21] Office for Human Research Protections (OHRP) (2017). 2018 Requirements (2018 Common Rule). U.S. Department of Health & Human Services.

[22] Ghebreyesus T (2020). WHO Director-General's opening remarks at the media briefing on COVID-19 - 11 March 2020. World Health Organization.

[23] Patel SY, Mehrotra A, Huskamp HA, Uscher-Pines L, Ganguli I, Barnett ML (2021). Trends in outpatient care delivery and telemedicine during the COVID-19 pandemic in the US. JAMA Intern Med.

[24] Vidal-Alaball J, Fernandez-Luque L, Marin-Gomez FX, Ahmed W (2019). A new tool for public health opinion to give insight into telemedicine: Twitter poll analysis. JMIR Form Res.

[25] The COVID-19 Healthcare Coalition Telehealth Workgroup (2021). Telehealth impact study: patient survey executive summary. The MITRE Corporation.

[26] (2020). Improving rural health and telehealth access. Federal Register - The Daily Journal of the United States Government.

[27] Shatzkes M, Kraus E, Rai K (2020). It’s official: telehealth benefits have been expanded for Medicare beneficiaries. The National Law Review.

[28] Latifi R, Doarn C (2020). Perspective on COVID-19: finally, telemedicine at center stage. Telemed J E Health.

[29] Assistant Secretary for Public Affairs (ASPA) (2020). Telehealth: delivering care safely during COVID-19. U.S. Department of Health & Human Services.

[30] Gore RJ, Diallo S, Padilla J (2015). You are what you tweet: connecting the geographic variation in America's obesity rate to Twitter content. PLoS One.

[31] Padilla JJ, Kavak H, Lynch CJ, Gore RJ, Diallo SY (2018). Temporal and spatiotemporal investigation of tourist attraction visit sentiment on Twitter. PLoS One.

[32] Perrin A, Anderson M (2019). Share of U.S. adults using social media, including Facebook, is mostly unchanged since 2018. Pew Research Center.

