# Supplementary material for: Language and Sentiment Regarding Telemedicine and COVID-19 on Twitter: Longitudinal Infodemiology Study
Source: J Med Internet Res. 2021 Jun 21;23(6):e28648. doi: 10.2196/28648 (PMC8218898; doi:10.2196/28648)
Supplement: Multimedia Appendix 1 [file jmir_v23i6e28648_app1.docx]

**Appendix Material 1.**

Telemedicine and COVID-19 search terms used to select tweets. Words were derived from prior literature (see [11]) as well as the National Institutes of Health National Library of Medicine Medical Subject Headings thesaurus (see [15]). Note that “virtual care” and “virtualcare” were removed because they produced results unrelated to telemedicine (e.g., “virtual career fair”).

| Telemedicine Terms | COVID-19 Terms |
| --- | --- |
| - "telemedicine" - "telehealth" - "tele-health" - "tele health" - "televisit" - "tele-visit" - "tele visit" - "virtual healthcare" - "virtualhealthcare" - "tele medicine" - "telemed" - "TelemedNow" - "digital health" - "digitalhealth" - "digihealth" - "remote monitoring" - "remotemonitoring" - "connected health" - "connectedhealth" - "mobile health" - "mhealth" - "ehealth" - "m-health" - "e-health" | - "COVID 19" - "2019 nCoV" - "Coronavirus Disease 19" - "2019 Novel Coronavirus" - "COVID19" - "Coronavirus Disease 2019" - "SARS Coronavirus 2 Infection" - "SARS CoV 2" |
