# Supplementary material for: Language and Sentiment Regarding Telemedicine and COVID-19 on Twitter: Longitudinal Infodemiology Study
Source: J Med Internet Res. 2021 Jun 21;23(6):e28648. doi: 10.2196/28648 (PMC8218898; doi:10.2196/28648)
Supplement: Multimedia Appendix 2 [file jmir_v23i6e28648_app2.docx]

**Appendix Material 2.**

Zero-inflated Poisson models

| Table S1: Association Between Month and Retweets, Telemedicine Model Only | | | | |
| --- | --- | --- | --- | --- |
|  | Poisson Model | | Zero-Inflated Model | |
| Parameter | IRR^a^ (95% CI^b^) | *P* value^c^ | OR^d^ (95% CI) | *P* value^c^ |
| Month (Reference: March 2020)  January 2020  February 2020  April 2020  May 2020  June 2020  July 2020  August 2020  September 2020  October 2020  November 2020  December 2020  January 2021  February 2021  March 2021  April 2021 | 3.04 (0.53, 17.5)  1.05 (0.67, 1.64)  0.73 (0.59, 0.91)  0.68 (0.55, 0.84)  0.91 (0.58, 1.44)  0.74 (0.59, 0.93)  0.77 (0.57, 1.05)  0.55 (0.43, 0.71)  0.59 (0.45, 0.77)  0.84 (0.43, 1.63)  0.51 (0.41, 0.64)  0.69 (0.51, 0.93)  0.63 (0.49, 0.81)  0.75 (0.58, 0.97)  0.76 (0.59, 0.98) | .213  .826  .005  < .001  .695  .009  .100  < .001  < .001  .609  < .001  .015  < .001  .029  .035 | 0.77 (0.42, 1.39)  0.88 (0.79, 0.99)  1.11 (1.08, 1.14)  1.18 (1.14, 1.21)  1.24 (1.20, 1.28)  1.23 (1.19, 1.27)  1.58 (1.53, 1.64)  1.32 (1.27, 1.37)  1.42 (1.37, 1.47)  1.39 (1.34, 1.44)  1.29 (1.24, 1.34)  1.08 (1.04, 1.12)  0.98 (0.94, 1.01)  1.03 (0.99, 1.07)  1.41 (1.36, 1.47) | .383  .031  < .001  < .001  < .001  < .001  < .001  < .001  < .001  < .001  < .001  < .001  .198  .108  < .001 |
| ^a^ IRR: Incidence Rate Ratio  ^b^ CI: Confidence Interval  ^c^ *P* values calculated from zero-inflated Poisson using robust standard error estimates (Poisson portion only)  ^d^ OR: Odds Ratio | | | | |

| Table S2: Association Between Month and Retweets | | | | |
| --- | --- | --- | --- | --- |
|  | Poisson Model | | Zero-Inflated Model | |
| Parameter | IRR^a^ (95% CI^b^) | *P* value^c^ | OR^d^ (95% CI) | *P* value^c^ |
| Month (Reference: March 2020)  January 2020  February 2020  April 2020  May 2020  June 2020  July 2020  August 2020  September 2020  October 2020  November 2020  December 2020  January 2021  February 2021  March 2021  April 2021 | 0.48 (0.22, 1.04)  2.26 (0.83, 6.18)  1.11 (0.90, 1.38)  1.27 (0.87, 1.84)  1.34 (0.82, 2.19)  3.81 (1.48, 9.82)  1.13 (0.94, 1.37)  1.46 (0.96, 2.24)  3.79 (1.21, 11.9)  1.13 (0.86, 1.49)  0.84 (0.70, 1.01)  1.00 (0.81, 1.24)  0.70 (0.59, 0.83)  1.33 (0.81, 2.17)  1.06 (0.82, 1.36) | .062  .111  .326  .220  .245  .006  .193  .080  .022  .383  .064  .988  < .001  .263  .659 | 0.75 (0.37, 1.50)  0.83 (0.74, 0.93)  1.06 (1.03, 1.09)  1.09 (1.05, 1.12)  1.08 (1.05, 1.12)  1.16 (1.12, 1.20)  1.14 (1.10, 1.18)  1.18 (1.14, 1.23)  1.13 (1.09, 1.18)  1.14 (1.10, 1.19)  1.13 (1.09, 1.17)  1.04 (1.00, 1.08)  1.02 (0.98, 1.06)  1.02 (0.98, 1.06)  1.13 (1.08, 1.18) | .412  .002  < .001  < .001  < .001  < .001  < .001  < .001  < .001  < .001  < .001  .049  .379  .403  < .001 |
| Data Set (Reference: COVID)  Telemedicine | 0.79 (0.63, 0.99) | .038 | 0.63 (0.61, 0.65) | < .001 |
| Month * Data Set (Reference: COVID, March 2020)  Telemedicine, January 2020  Telemedicine, February 2020  Telemedicine, April 2020  Telemedicine, May 2020  Telemedicine, June 2020  Telemedicine, July 2020  Telemedicine, August 2020  Telemedicine, September 2020  Telemedicine, October 2020  Telemedicine, November 2020  Telemedicine, December 2020  Telemedicine, January 2021  Telemedicine, February 2021  Telemedicine, March 2021  Telemedicine, April 2021 | 6.35 (0.94, 43.0)  0.46 (0.15, 1.40)  0.66 (0.48, 0.89)  0.54 (0.35, 0.83)  0.68 (0.35, 1.33)  0.19 (0.07, 0.51)  0.68 (0.47, 0.98)  0.38 (0.23, 0.62)  0.16 (0.05, 0.50)  0.74 (0.36, 1.52)  0.61 (0.45, 0.81)  0.69 (0.47, 0.99)  0.90 (0.66, 1.21)  0.57 (0.32, 0.99)  0.72 (0.50, 1.03) | .058  .172  .007  .005  .264  .001  .038  < .001  .002  .420  .001  .046  .481  .045  .070 | 1.02 (0.41, 2.56)  1.06 (0.90, 1.25)  1.05 (1.01, 1.09)  1.09 (1.04, 1.13)  1.15 (1.10, 1.20)  1.07 (1.02, 1.12)  1.39 (1.32, 1.46)  1.12 (1.06, 1.18)  1.25 (1.19, 1.32)  1.21 (1.15, 1.28)  1.14 (1.09, 1.20)  1.03 (0.98, 1.09)  0.96 (0.91, 1.01)  1.01 (0.96, 1.07)  1.25 (1.18, 1.13) | .959  .453  .014  < .001  < .001  .007  < .001  < .001  < .001  < .001  < .001  .235  .127  .644  < .001 |
| ^a^ IRR: Incidence Rate Ratio  ^b^ CI: Confidence Interval  ^c^ *P* values calculated from zero-inflated Poisson using robust standard error estimates (Poisson portion only)  ^d^ OR: Odds Ratio | | | | |

| Table S3: Association Between Month and Favorites, Telemedicine Model Only | | | | |
| --- | --- | --- | --- | --- |
|  | Poisson Model | | Zero-Inflated Model | |
| Parameter | IRR^a^ (95% CI^b^) | *P* value^c^ | OR^d^ (95% CI) | *P* value^c^ |
| Month (Reference: March 2020)  January 2020  February 2020  April 2020  May 2020  June 2020  July 2020  August 2020  September 2020  October 2020  November 2020  December 2020  January 2021  February 2021  March 2021  April 2021 | 3.43 (0.53, 22.4)  0.74 (0.43, 1.28)  0.65 (0.44, 0.97)  0.63 (0.42, 0.94)  0.74 (0.44, 1.26)  0.58 (0.39, 0.86)  0.60 (0.38, 0.95)  0.46 (0.31, 0.70)  0.50 (0.33, 0.75)  0.73 (0.39, 1.34)  0.48 (0.32, 0.72)  0.59 (0.38, 0.91)  0.61 (0.39, 0.95)  0.62 (0.40, 0.94)  0.64 (0.42, 0.97) | .198  .280  .035  .022  .272  .007  .029  < .001  .001  .305  < .001  .018  .029  .024  .034 | 1.16 (0.64, 2.11)  1.13 (1.01, 1.26)  1.09 (1.06, 1.12)  1.17 (1.14, 1.21)  1.25 (1.21, 1.29)  1.26 (1.22, 1.30)  1.36 (1.31, 1.40)  1.37 (1.32, 1.42)  1.36 (1.32, 1.41)  1.32 (1.28, 1.37)  1.31 (1.27, 1.36)  1.16 (1.12, 1.21)  1.02 (0.98, 1.06)  1.07 (1.04, 1.03)  1.62 (1.55, 1.68) | .626  .039  < .001  < .001  < .001  < .001  < .001  < .001  < .001  < .001  < .001  < .001  .264  < .001  < .001 |
| ^a^ IRR: Incidence Rate Ratio  ^b^ CI: Confidence Interval  ^c^ *P* values calculated from zero-inflated Poisson using robust standard error estimates (Poisson portion only)  ^d^ OR: Odds Ratio | | | | |

| Table S4: Association Between Month and Favorites | | | | | | |  |
| --- | --- | --- | --- | --- | --- | --- | --- |
|  | Poisson Model | | | Zero-Inflated Model | | | |
| Parameter | IRR^a^ (95% CI^b^) | *P* value^c^ | OR^d^ (95% CI) | | *P* value^c^ |  |  |
| Month (Reference: March 2020)  January 2020  February 2020  April 2020  May 2020  June 2020  July 2020  August 2020  September 2020  October 2020  November 2020  December 2020  January 2021  February 2021  March 2021  April 2021 | 0.29 (0.15, 0.57)  1.09 (0.56, 2.13)  1.21 (0.91, 1.60)  1.83 (0.89, 3.78)  1.16 (0.89, 1.51)  4.96 (1.66, 14.8)  1.19 (0.97, 1.46)  1.60 (1.04, 2.47)  6.52 (1.68, 25.2)  1.38 (1.09, 1.74)  1.16 (0.91, 1.48)  1.21 (0.98, 1.50)  0.90 (0.71, 1.14)  2.98 (1.00, 8.88)  1.39 (0.97, 2.00) | < .001  .794  .187  .098  .282  .004  .094  .031  .007  .007  .241  .080  .381  .050  .073 | 1.73 (0.86, 3.51)  1.06 (0.94, 1.18)  1.19 (1.16, 1.23)  1.30 (1.26, 1.34)  1.36 (1.31, 1.40)  1.42 (1.37, 1.46)  1.49 (1.44, 1.54)  1.58 (1.53, 1.64)  1.46 (1.41, 1.52)  1.33 (1.29, 1.38)  1.35 (1.30, 1.40)  1.34 (1.30, 1.40)  1.30 (!.26, 1.36)  1.30 (1.25, 1.35)  1.46 (1.40, 1.52) | | .126  .346  < .001  < .001  < .001  < .001  < .001  < .001  < .001  < .001  < .001  < .001  < .001  < .001  < .001 |  |  |
| Data Set (Reference: COVID)  Telemedicine | 0.86 (0.58, 1.28) | .450 | 0.85 (0.83, 0.88) | | < .001 |  |  |
| Month * Data Set (Reference: COVID, March 2020)  Telemedicine, January 2020  Telemedicine, February 2020  Telemedicine, April 2020  Telemedicine, May 2020  Telemedicine, June 2020  Telemedicine, July 2020  Telemedicine, August 2020  Telemedicine, September 2020  Telemedicine, October 2020  Telemedicine, November 2020  Telemedicine, December 2020  Telemedicine, January 2021  Telemedicine, February 2021  Telemedicine, March 2021  Telemedicine, April 2021 | 11.7 (1.60, 85.2)  0.68 (0.28, 1.61)  0.54 (0.33, 0.88)  0.34 (0.15, 0.78)  0.64 (0.35, 1.16)  0.12 (0.04, 0.37)  0.50 (0.30, 0.83)  0.29 (0.16, 0.52)  0.08 (0.01, 0.31)  0.53 (0.27, 1.01)  0.41 (0.26, 0.66)  0.48 (0.30, 0.79)  0.67 (0.41, 1.12)  0.21 (0.06, 0.67)  0.46 (0.27, 0.80) | .015  .374  .013  .010  .143  < .001  .008  < .001  < .001  .055  < .001  .004  .124  .008  .005 | 0.67 (0.27, 1.69)  1.07 (0.91, 1.25)  0.91 (0.88, 0.95)  0.90 (0.87, 0.94)  0.92 (0.88, 0.96)  0.89 (0.85, 0.93)  0.91 (0.87, 0.96)  0.86 (0.82, 0.91)  0.93 (0.89, 0.98)  0.99 (0.94, 1.04)  0.97 (0.93, 1.02)  0.86 (0.82, 0.91)  0.78 (0.74, 0.83)  0.82 (0.78, 0.87)  1.11 (1.05, 1.17) | | .395  .427  < .001  < .001  < .001  < .001  < .001  < .001  .004  .785  .240  < .001  < .001  < .001  < .001 |  |  |
| ^a^ IRR: Incidence Rate Ratio  ^b^ CI: Confidence Interval  ^c^ *P* values calculated from zero-inflated Poisson using robust standard error estimates (Poisson portion only)  ^d^ OR: Odds Ratio | | | | | | |  |
