# Supplementary material for: Language and Sentiment Regarding Telemedicine and COVID-19 on Twitter: Longitudinal Infodemiology Study
Source: J Med Internet Res. 2021 Jun 21;23(6):e28648. doi: 10.2196/28648 (PMC8218898; doi:10.2196/28648)

**Appendix Material 3.**

Sentiment variations between the telemedicine-COVID data set and the general-COVID data set by month. The telemedicine-COVID data set had a significantly higher proportion of positive tweets compared to the general-COVID data set in March 2020 (54.4% vs. 40.7%, *P* < .001); April 2020 (52.8% vs. 41.0%, *P* < .001); May 2020 (54.1% vs. 39.4%, *P* < .001); June 2020 (51.8% vs. 39.3%, *P* < .001); July 2020 (50.9% vs. 37.6%, *P* < .001); August 2020 (58.6% vs. 38.3%, *P* < .001); September 2020 (55.8% vs. 36.3%, *P* < .001); October 2020 (48.9% vs. 37.6%, *P* < .001); November 2020 (48.4% vs. 38.9%, *P* < .001); December 2020 (48.4% vs. 38.6%, *P* < .001); January 2021 (47.0% vs. 36.1%, *P* < .001); February 2021 (47.6% vs. 36.4%, *P* < .001); March 2021 (44.7% vs. 36.9%, *P* < .001); and April 2021 (40.4% vs. 33.3%, *P* < .001).


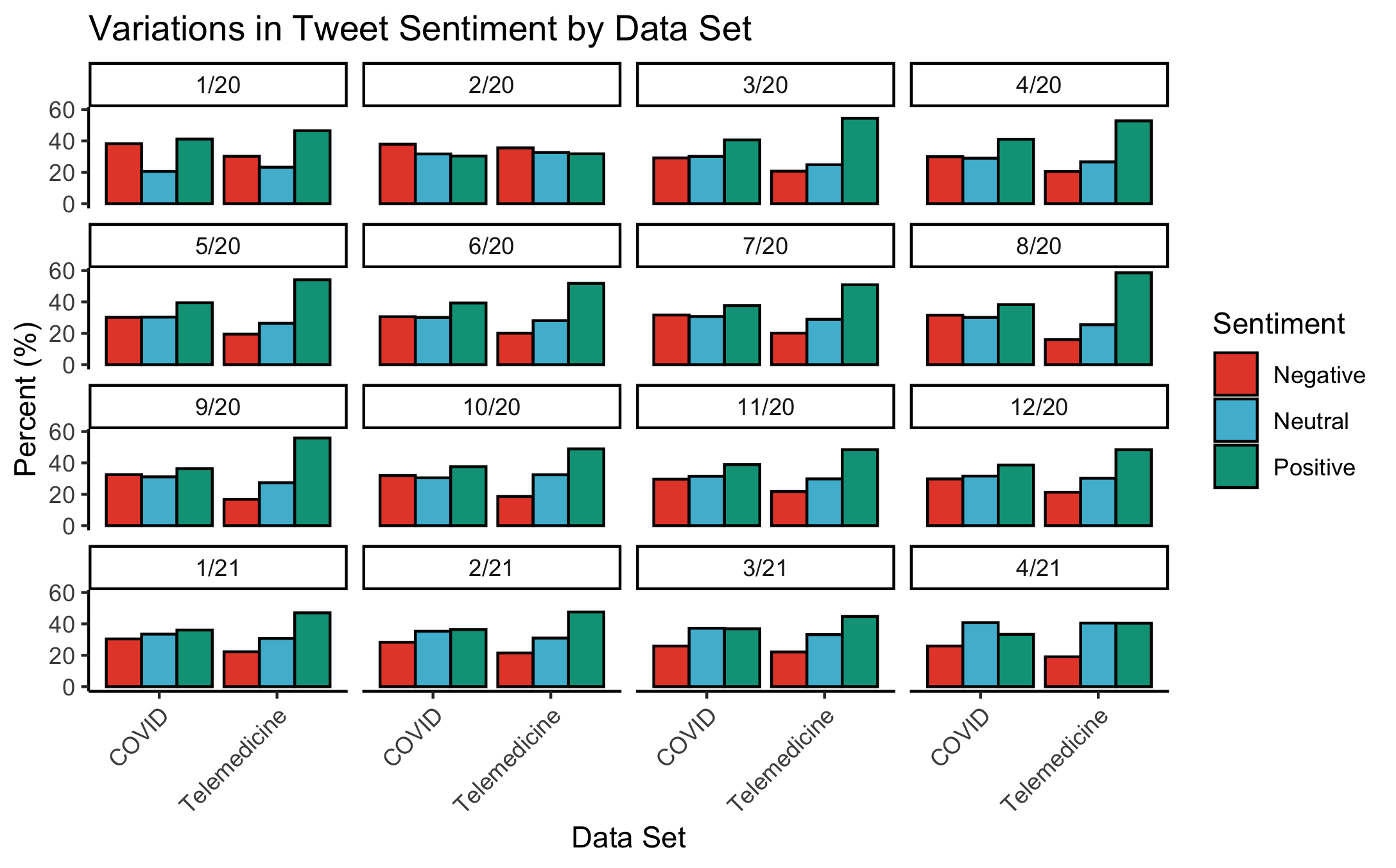

Supplement: Multimedia Appendix 3 [file jmir_v23i6e28648_app3.docx]
