# Supplementary material for: Language and Sentiment Regarding Telemedicine and COVID-19 on Twitter: Longitudinal Infodemiology Study
Source: J Med Internet Res. 2021 Jun 21;23(6):e28648. doi: 10.2196/28648 (PMC8218898; doi:10.2196/28648)

**Appendix Material 4.**

Word clouds of unigrams for the telemedicine-specific COVID-19 tweets (top) and the general COVID-19 sample tweets (bottom).


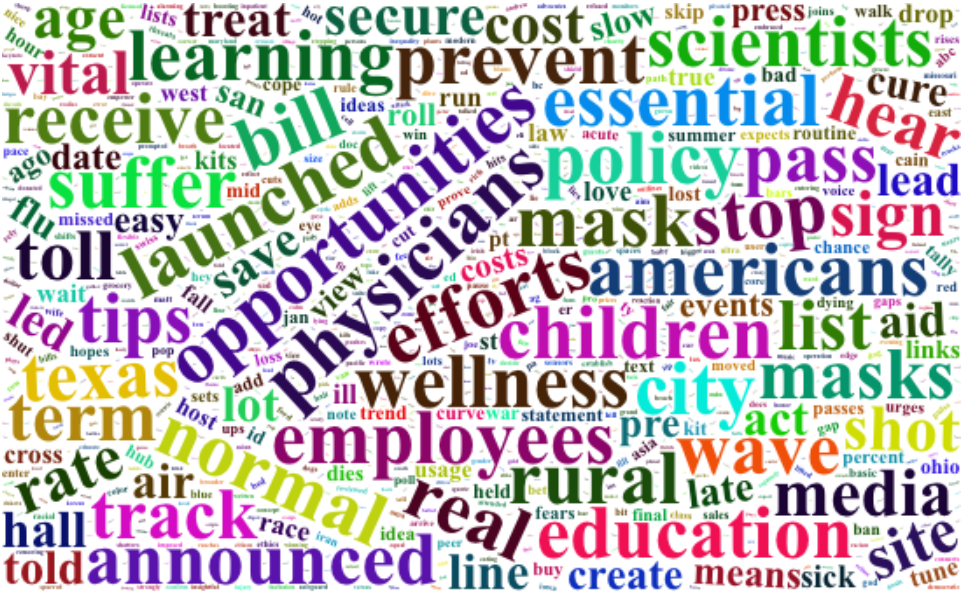


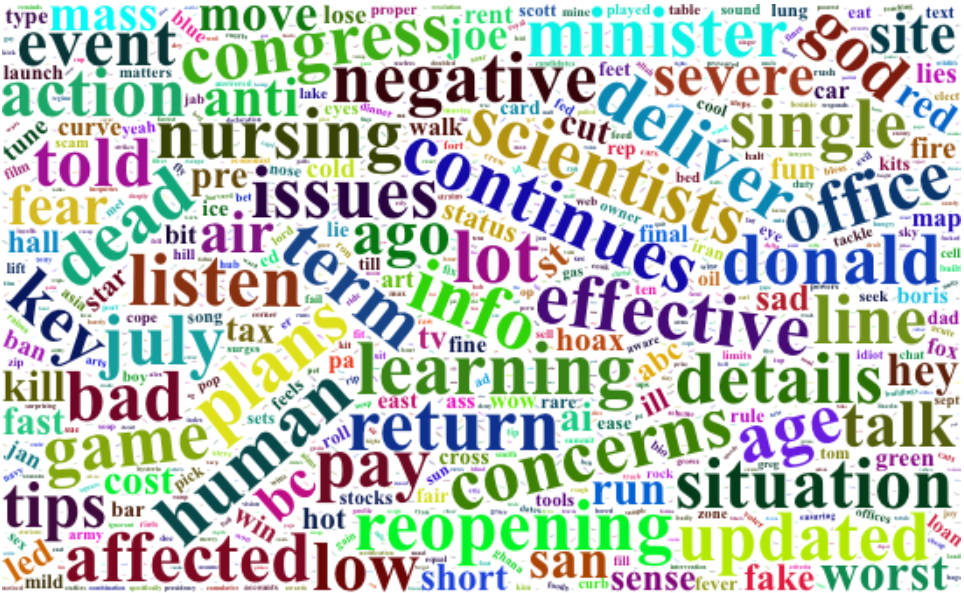

Supplement: Multimedia Appendix 4 [file jmir_v23i6e28648_app4.docx]
